# Production of hepatitis E virus-like particles presenting multiple foreign epitopes by co-infection of recombinant baculoviruses

**DOI:** 10.1038/srep21638

**Published:** 2016-02-24

**Authors:** Ryoichi Shima, Tian Cheng Li, Yutaka Sendai, Chikako Kataoka, Yoshio Mori, Takayuki Abe, Naokazu Takeda, Toru Okamoto, Yoshiharu Matsuura

**Affiliations:** 1 Department of Molecular Virology, Research Institute for Microbial Diseases, Osaka University, Osaka , Japan; 2 Central Research Institute for Feed and Livestock, ZEN-NOH (National Federation of Agricultural Co-operative Associations), Ibaraki, Japan; 3 Department of Virology II, National Institute of Infectious Diseases, Tokyo, Japan; 4 Department of Virology III, National Institute of Infectious Diseases, Tokyo, Japan; 5 Thailand-Japan Research Collaboration Center on Emerging and Re-emerging Infections (RCC-ERI), Nonthaburi, Thailand

## Abstract

Hepatitis E virus (HEV) causes not only endemics via a fecal-oral route but also sporadic cases via zoonotic transmission or blood transfusion. HEV-like particles (HEV-LP) produced by using a baculovirus expression system are considered a candidate for mucosal vaccines for HEV infection. In this study, we attempted to produce a chimeric HEV-LP presenting various foreign epitopes on its surface. Expression of the recombinant capsid proteins carrying a myc- or FLAG-tag inserted between amino acid residues 488 and 489, which are located in the exterior loop on the protruding domain of the HEV capsid, resulted in the production of recombinant HEV-LP. Although expression of the recombinant capsid protein carrying the HA-tag inserted at the same site failed to produce any particles, co-expression with the myc-tagged capsid protein successfully yielded a chimeric HEV-LP consisting of both recombinant capsid proteins. Immunoprecipitation analyses confirmed that the chimeric particles present these foreign epitopes on the surface. Similar results were obtained for the expression of the recombinant capsid proteins carrying neutralizing epitopes of Japanese encephalitis virus. These results suggest the chimeric HEV-LP system provides a novel vaccine carrier that can accommodate multiple neutralizing epitopes on its surface.

Hepatitis E, which is caused by hepatitis E virus (HEV), is an endemic disease in developing countries of Asia, Africa and Latin America^1^. HEV is transmitted primarily by a fecal-oral route through drinking water and foods contaminated with HEV in countries where sanitation is suboptimal^1^,^2^,^3^,^4^,^5^,^6^,^7^,^8^,^9^. However, sporadic cases have been reported in non-endemic areas, including some developed countries. Epidemiological studies have reported that viral genome or serum antibodies against HEV were detected in domestic and wild animals worldwide even in developed countries^10^,^11^,^12^,^13^,^14^. In Japan, HEV may be food-borne, as suggested by the presence of HEV in pig liver intended for human consumption^14^. Indeed, domestic pigs show particularly high prevalence of HEV antibodies, and several cases of acute hepatitis E have been epidemiologically linked to eating undercooked pig liver and meat^15^. Collectively, these findings provide clear evidence of food-borne zoonotic transmission of HEV and highlight the need for safety measures in the production of pork. Genotypes 1 and 2 of HEV are restricted to humans and responsible for the endemic cases in developing countries, while genotypes 3 and 4 are found in the zoonotic cases^16^.

HEV is characterized as a non-enveloped RNA virus and is the sole member of *Hepevirus* within the family *Hepeviridae*^17^. HEV has a single-stranded and positive-sense RNA genome of approximately 7.2 kb in length that possesses a short 5′ untranslated region (UTR), three open reading frames (ORF) and a short 3′ UTR^18^. ORF1, 2 and 3 encode large nonstructural proteins containing motifs of methyltransferase, polymerase, papain-like protease and helicase, viral capsid protein, and a small phosphorylated protein with multiple functions, respectively^19^,^20^,^21^,^22^. Expression of the amino acid residues 112–608 of the capsid protein in insect cells by a baculovirus system resulted in the formation of self-assembled HEV-like particles (HEV-LP) and release of a large amount of HEV-LP into the culture supernatants^20^,^23^. Cryo-electron microscopic analysis and X-ray crystallography have revealed that HEV-LP is a T = 1 icosahedral particle composed of 60 copies of the capsid protein^22^,^23^. HEV-LP appeared to be empty because it exhibited no significant density for RNA and because it was 270 Å in diameter, which is less than the diameter of partially purified native virions^22^,^23^. The truncated capsid protein is structurally divided into three domains, the shell (S), middle (M), and protruding (P) domains. The dimer of the P domain forms the prominent protrusion on the surface of HEV-LP and is proposed to possess binding regions for not-yet-known receptors and epitopes to neutralizing antibodies^24^.

Oral administration of HEV-LP without adjuvant induced systemic and mucosal immune responses against HEV in mice and protective immunity in monkeys^25^. These results indicated that HEV-LP has a high immunogenicity in the absence of adjuvants, suggesting it would be a promising candidate for an effective oral vaccine without the requirement of an adjuvant^25^. Furthermore, Niikura *et al*. showed that a chimeric HEV-LP formed with a capsid protein fused with a foreign epitope at the C-terminus exhibited immunogenicity to the foreign epitopes^26^, suggesting that HEV-LP may also have potential as vaccine carriers of foreign epitopes.

In this study, we chose the large exterior loop of the P domain of the capsid protein as the insertion site of foreign epitopes, because a previous study on the crystal structure of HEV-LP suggested that this region was permissive for manipulation^19^. Expression of the recombinant HEV capsid proteins carrying a myc-tag or FLAG-tag but not an HA-tag inserted in the exterior loop on the P domain resulted in the production of a large amount of the recombinant HEV-LP that was comparable to the quantity of wild-type HEV-LP. Interestingly, co-expression of the capsid proteins carrying an HA-tag with those carrying a myc-tag successfully yielded a chimeric HEV-LP containing these recombinant capsid proteins. Thus our novel system for producing chimeric HEV-LP presenting multiple foreign epitopes on the surface might be an ideal platform for a multivalent mucosal vaccine.

## Materials and Methods

### Cells

Sf9 cells derived from *Spodoptera frugiperda* were grown in SF900-II medium (Life Technologies, Carlsbad, CA) supplemented with or without 10% fetal bovine serum (FBS) (Sigma, St. Louis, MO) at 27 °C. Tn5 cells derived from *Trichoplusia ni* were grown in EXCELL 405 serum-free medium (JRH, Lenexa, KS) at 27 °C.

### Antibodies

Antibodies to c-myc- (M4439), HA- (H3668), and FLAG (M5, F4042)-tags were purchased from Sigma. Rabbit antibodies against HEV-LP^10^ and peptides corresponding to Japanese encephalitis virus (JEV) E protein epitopes, amino acid residues from 337 to 345 (JEV1 epitope) and amino acids from 362 to 369 (JEV2 epitope) were prepared by Scrum Inc. (Tokyo, Japan).

### Construction of recombinant baculoviruses

**Figure 1:**
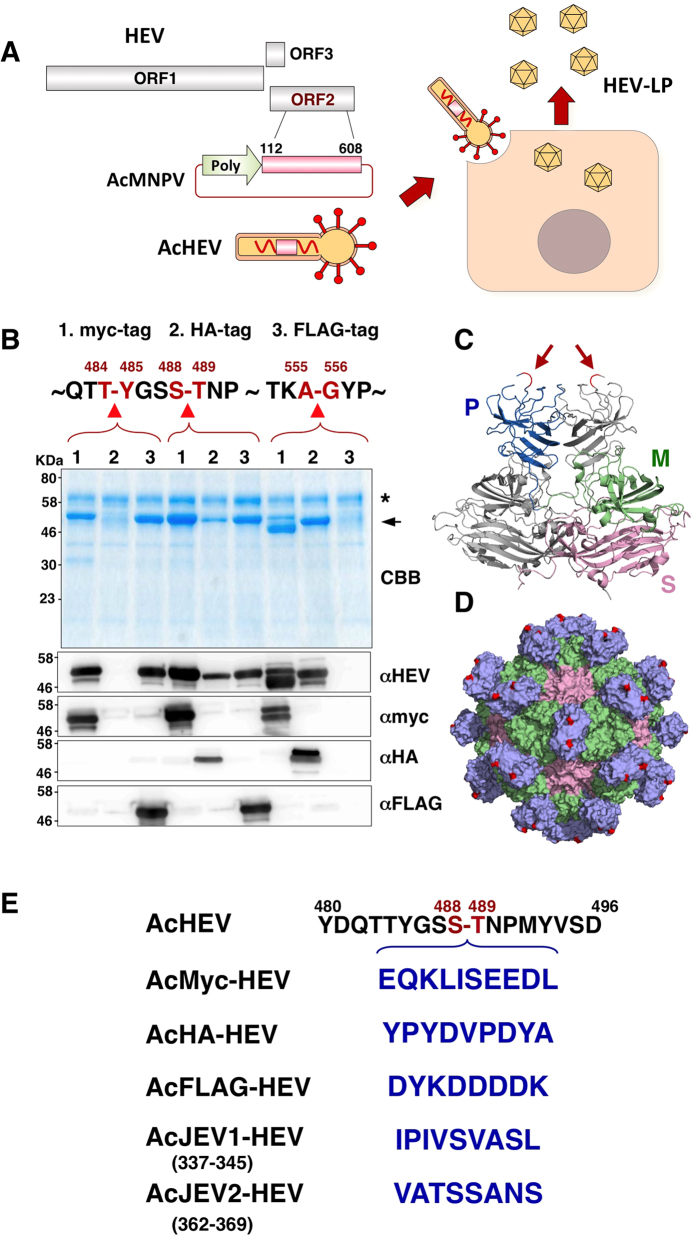
(**A**) Production of HEV-LP in insect cells by infection with AcHEV, a recombinant baculovirus possessing truncated ORF2 (amino acid residues from 112 to 608) of HEV under the polyhedrin promoter. (**B**) Expression of the truncated HEV capsid proteins harboring myc- (1), HA- (2), or FLAG- (3) tagg in the three insertion sites indicated on the top. The recombinant capsid proteins secreted into the culture media were detected by CBB staining and immunoblotting. Asterisk and arrow in the CBB staining gel indicate bovine serum albumin and recombinant HEV capsids, respectively. (**C**) The ribbon diagram of crystal structures of capsid protein dimers of HEV-LP. The S (shell, amino acid residues from 129 to 319), M (middle, amino acid residues from 320 to 455) and P (protruding, amino acid residues from 456 to 606) domains of one monomer are shown in pink, green and blue, respectively. The insertion sites for foreign epitopes in the P domains of the capsid dimers were shown by red lines and arrows. (**D**) Three-dimensional representation of HEV-LP and the insertion sites of foreign epitopes are shown in red. (**E**) Recombinant baculoviruses used in this study and amino acid residues of the epitopes inserted into HEV capsid. AcHEV, AcMyc-HEV, AcHA-HEV, AcFLAG-HEV, AcJEV1-HEV, and AcJEVE2-HEV are recombinant baculoviruses expressing wild-type HEV capsid, recombinant capsids harboring myc-, HA-, FLAG, JEV1, and JEV2-sequences between Ser^488^ and Thr^489^ of HEV capsid, respectively.

The recombinant baculovirus *Autographa californica* multiple nucleopolyhedrovirus (AcMNPV) encoding amino acid residues 112 to 608 of the ORF2 of the HEV genotype 3, 2712 strain (a wild-type capsid protein) was prepared as described previously^20^,^23^ (Fig. 1A). The cDNAs of foreign epitopes, i.e., c-myc-, HA- and FLAG-tags or JEV E epitopes, were inserted between amino acid residues 485 and 486, 488 and 489, or 555 and 556 by the method of insertional mutation by overlap extension^27^ using the primers shown in Table 1. Briefly, in the first PCR, the front parts of ORF2 were amplified by PCR using a forward primer, HEV (334) /BamHI, and reverse primers, HEV (1452)-c-myc/Rv, HEV (1452)-HA/Rv, HEV (1452)-FLAG/Rv, HEV (1464)-c-myc/Rv, HEV (1464)-HA/Rv, HEV (1464)-FLAG/Rv, HEV (1464)-JE (337)/Rv, HEV (1464)-JE (362)/Rv, HEV (1665)-c-myc/Rv, HEV (1665)-HA/Rv, or HEV (1665)-FLAG/Rv. In the second PCR, the backward parts of ORF2 were amplified by PCR using forward primers, c-myc-HEV (1453)/Fw, HA-HEV (1453)/Fw, FLAG-HEV (1453)/Fw, c-myc-HEV (1465)/Fw, HA-HEV (1465)/Fw, FLAG-HEV (1465)/Fw, JE (337)-HEV (1465)/Fw, JE (362)-HEV (1465)/Fw, c-myc-HEV (1666)/Fw, HA-HEV (1666)/Fw, or FLAG-HEV (1666)/Fw, and a reverse primer, HEV (1824)/XhoI/Rv. And then, in the final PCR, using both of the corresponding front and backward parts as templates, the whole genes of the truncated capsid protein inserted with foreign epitopes were amplified with a forward primer HEV (334)/BamHI/Fw and a reverse primer HEV (1824)/XhoI/Rv. The amplified genes were introduced into the pFastBac1 plasmid. The resulting plasmids were transformed into DH10Bac strain and white colonies of bacteria were selected on plates containing kanamycin (50 μg/ml, Sigma), gentamycin (7 μg/ml, Sigma) and tetracyclin (10 μg/ml, Sigma). Bacmid DNAs derived from the white colonies were extracted by QIAprep Spin Miniprep Kit (Qiagen) following manufacture’s protocol and transfected into Sf9 cells by using the Unifector Reagent (B-Bridge, Sunnyvale, CA) according to the manufacturer’s instruction. Recombinant baculoviruses recovered from the culture medium (passage 1) were propagated in Sf9 cells and determined infectious titers by using plaque assay as previously reported^28^.

### Preparation of HEV-LP

To produce the wild-type and chimeric HEV-LP, Tn5 cells were infected with recombinant baculoviruses at a multiplicity of infection (moi) of 5. The culture media were harvested at 7 days post-infection. The intact cells, cell debris and progeny baculoviruses were removed by centrifugation at 10,000 × g for 40 min. The supernatants were further centrifuged at 100,000 × g for 3 h. The resulting pellets suspended in phosphate buffered saline (PBS) overnight were centrifuged on a 10–40% sucrose density gradient at 38,000 × g for 2 h. The HEV-LP were recovered from middle fractions of the sucrose density gradient. Finally, the collected HEV-LP were dialyzed and concentrated by ultrafiltration using an Amicon Ultra-15 100K (Merck Millipore, Billerica, MA).

### SDS-PAGE and immunoblotting

The protein samples were subjected to 10–20% gradient sodium dodecyl sulfate-polyacrylamide gel electrophoresis (SDS-PAGE). The proteins were stained by Coomassie Brilliant Blue (CBB; Nacalai Tesque, Kyoto, Japan) or transferred to an Immobilon-P Transfer Membrane (Merck Millipore) and reacted with the appropriate antibodies. The immune complexes were visualized with the SuperSignal West Femto substrate (Thermo Scientific, Rockford, IL) and detected by an LAS-4000 image analyzer system (Fujifilm, Tokyo, Japan).

### Immunoprecipitation

The wild-type and chimeric HEV-LP were incubated with the appropriate antibodies and Protein G Sepharose 4 Fast Flow beads (GE Healthcare Japan, Tokyo, Japan). The immuno-complexes were precipitated with the beads by centrifugation at 14,000 × g for 30 sec and then were washed five times with lysis buffer (20 mM Tris-HCl [pH7.4], 135 mM NaCl, 10% glycerol, 1% TritonX-100). The proteins bound to the beads were boiled in 30 μl of SDS-PAGE loading buffer and then were analyzed by SDS-PAGE followed by immunoblotting.

### Electron microscopy

The HEV-LP purified by sucrose density gradient were mixed with 1.96 g of CsCl, and centrifuged at 35,000 rpm for 24 h at 4 °C in a Beckman SW50.1 rotor. The visible white band (at a density of 1.285 g/ml) was harvested by puncturing the tubes with a 21-gauge needle, diluted with EX-CELL 405 medium, and then centrifuged again in a Beckman TLA45 rotor at 45,000 rpm (125,000 × g) for 2 h to remove CsCl. The HEV-LP were placed on a carbon-coated grid, and the proteins were allowed to absorb into the grid for 5 min. After being rinsed with distilled water, the sample was stained with a 1% aqueous uranyl acetate solution and examined with a Hitachi H-7000 electron microscope (Hitachi High-Technologies, Tokyo, Japan). The chimeric HEV-LP were fixed on the grid, blocked with PBS containing 0.1% bovine serum albumin (PBS-BSA) at room temperature for 10 min, and then incubated with the c-myc- or HA-tagged antibody at room temperature for 15 min. After washing with PBS-BSA, the chimeric HEV-LP were incubated with gold-labeled second antibody at room temperature for 10 min and stained with 0.5% Uran for 5 sec after washing with PBS and distilled water.

## Results

### Selection of insertion site for foreign epitopes into the HEV capsid protein

Based on the three-dimensional information of the HEV-LP crystal structure, we selected three insertion sites for the foreign epitopes between amino acid residues 484 and 485, 488 and 489, and 555 and 556, which were located in a large loop within the P domain (Fig. 1B,C). These sites were likely to have at least two advantages for the insertion of foreign epitopes: first, the inserted epitopes would likely be displayed on the surface of the particle because this region is located in the outermost area of the particle (Fig. 1D); and second, the insertion of foreign epitopes was expected to have a minimal impact on the particle formation because this region is apart from any protein-protein interacting faces for the capsid oligomerization. To determine the suitable insertion site for production of the recombinant HEV-LP displaying foreign epitopes, a myc-, HA-, or FLAG-tag was inserted into these sites (Fig. 1B) and expressed in insect cells by using a baculoviral expression system. The myc-tagged capsid protein was secreted into the culture supernatants irrespective of the insertion sites, while expression of the HA- and FLAG-tagged capsid proteins inserted between amino acid residues 484/485 and 555/556, respectively, was not detected by immunoblot analyses. We therefore selected the amino acid residues 488/489 as an insertion site of foreign epitopes in this study (Fig. 1E).

### Production of chimeric HEV-LP possessing monovalent foreign epitope

**Figure 2:**
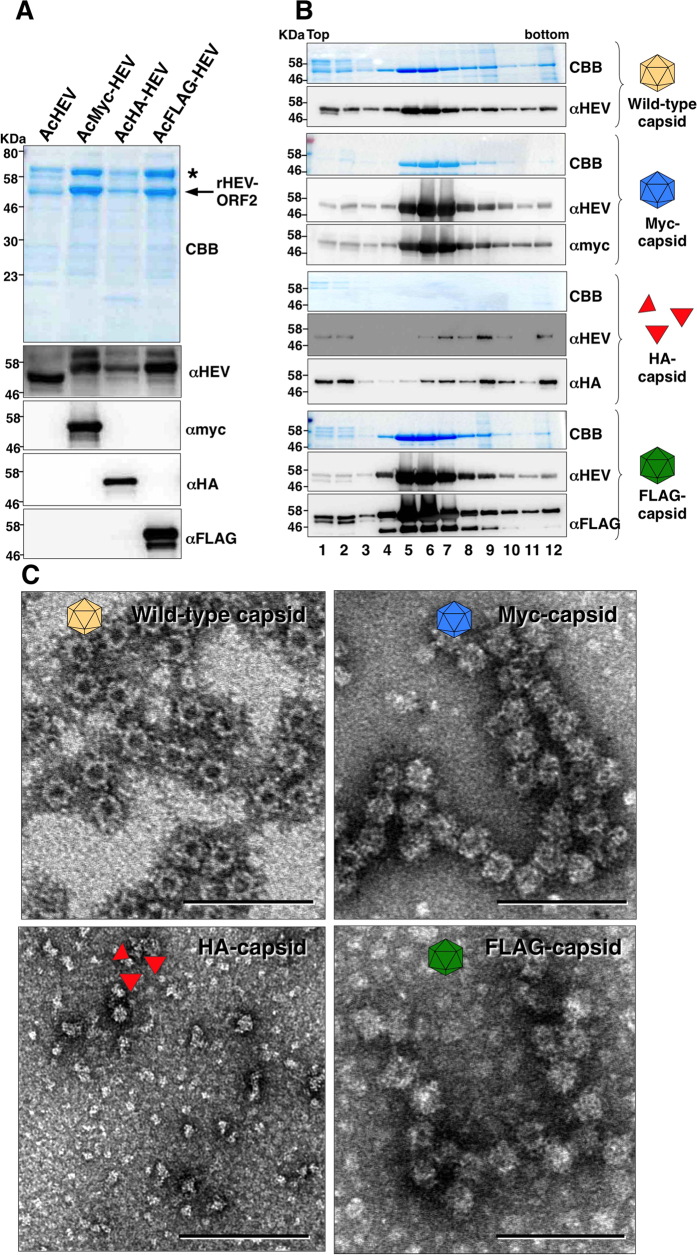
Production of chimeric HEV-LP possessing monovalent foreign epitope. (**A**) CBB staining and immunoblotting of the culture supernatants of Tn5 cells infected with AcHEV (light brown), AcMyc-HEV (blue), AcHA-HEV (red) or AcFLAG-HEV (green). Asterisk and arrow in the CBB staining gel indicate bovine serum albumin and recombinant HEV capsids, respectively. (**B**) Sucrose density gradient fractionation of the culture supernatants of cells infected with the recombinant viruses. Recombinant capsid proteins were indicated in the left of the panels. Each fractions were subjected to CBB staining and immunoblotting after SDS-PAGE. (**C**) Electron microscopic observation of the middle fractions of the sucrose density gradient. HEV-LP were imaged by negative staining. Bars indicate 100 nm.

Next, to determine the particle formation of the recombinant capsid proteins, the culture supernatants of Tn5 cells infected with the recombinant baculoviruses were concentrated by ultra-centrifugation and subjected to the sucrose density gradient. The wild-type HEV capsid and the recombinant capsids carrying the myc-, HA-, or FLAG-tag between amino acid residues 488/489 were detected in the culture supernatants of Tn5 cells infected with the recombinant viruses (Fig. 2A). After sucrose density gradient centrifugation, wild-type and recombinant HEV-LP possessing a myc- or FLAG-tag were recovered in the middle fractions (#5 to #8 fractions, Fig. 2B). Electron microscopic observation revealed that particles morphologically similar to the wild-type HEV-LP were detected in these fractions of the recombinant capsids harboring the myc- or FLAG-tag (Fig. 2C), demonstrating that the myc- and FLAG-tagged capsid proteins were able to form recombinant HEV-LP. In contrast, the HA-tagged capsid protein was not concentrated in the middle fractions by sucrose density gradient (Fig. 2B) and no HEV-LP was detected by electron microscopy (Fig. 2C). These results suggest that the particle formation of the recombinant HEV capsid proteins harboring foreign epitopes in the P domain was dependent on the amino acid sequences of the inserts.

### Production of chimeric HEV-LP possessing divalent foreign epitopes

**Figure 3:**
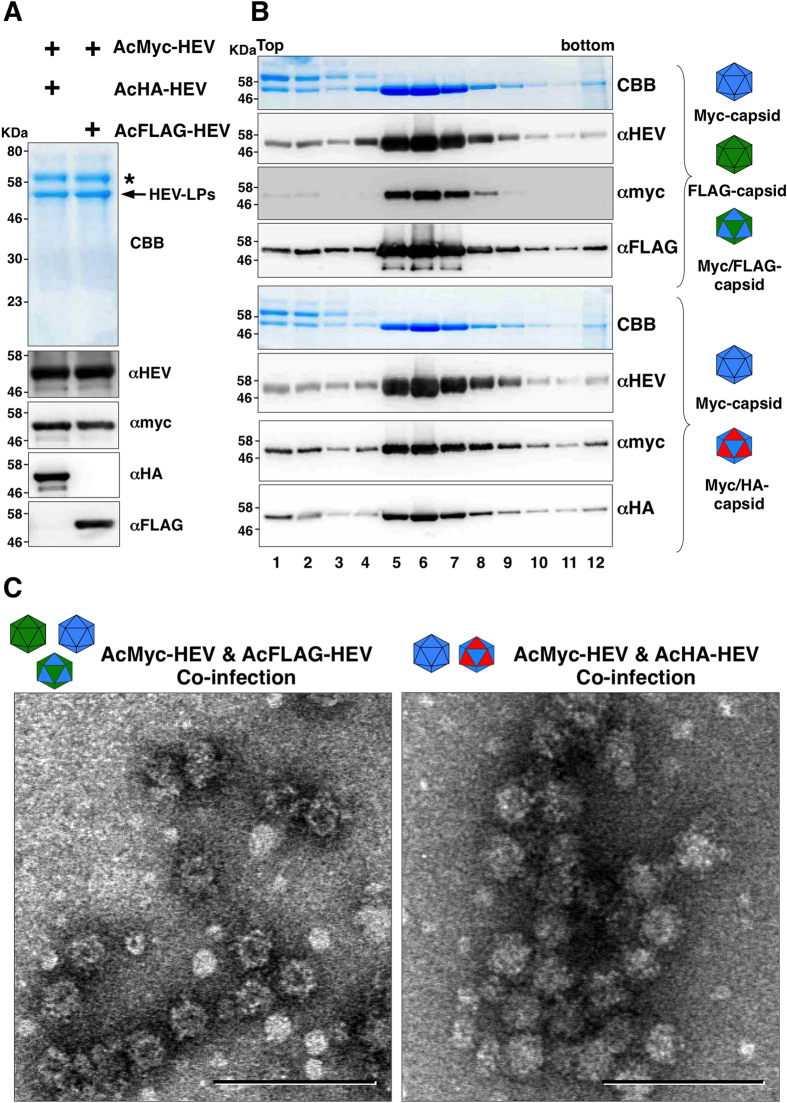
Production of chimeric HEV-LP possessing divalent foreign epitopes. (**A**) CBB staining and immunoblotting of the culture supernatants of Tn5 cells infected with AcMyc-HEV (blue) together with either AcHA-HEV (red) or AcFLAG-HEV (green). Asterisk and arrow in the CBB staining gel indicate bovine serum albumin and recombinant HEV capsids, respectively. (**B**) Sucrose density gradient fractionation of the culture supernatants of cells co-infected with the recombinant viruses. Putative recombinant and chimeric HEV-LP were indicated in the left of the panels. Each fractions were subjected to CBB staining and immunoblotting after SDS-PAGE. (**C**) Electron microscopic observation of the middle fractions of the sucrose density gradient. HEV-LP were imaged by negative staining. Bars indicate 100 nm.

It is known that a single T = 1 HEV-LP is composed of 60 copies of capsid protein^24^, and we therefore assumed that co-expression of the capsid proteins inserted with various foreign epitopes would result in the production of a chimeric HEV-LP displaying polyvalent foreign epitopes on the surface. To examine this possibility, the myc-tagged capsid protein was co-expressed with either the FLAG- or HA-tagged capsid protein by co-infection of the recombinant baculoviruses into insect cells. The recombinant capsid proteins were secreted in the culture supernatants and shown to retain the corresponding epitope tags by immunoblot analyses (Fig. 3A). The sucrose density gradient ultra-centrifugation assay followed by electron microscopic observation revealed that the chimeric HEV-LP consisting of the myc- and FLAG-tagged capsid proteins were generated upon co-expression (Fig. 3B,C). Surprisingly, an HA-tagged capsid protein incapable of producing particles via single expression (Fig. 2B,C) was shown to be incorporated into HEV-LP by co-expression with the myc-tagged capsid protein and concentrated in the middle fractions in the sucrose density gradient (Fig. 3B), and a large number of HEV-LP were detected by electron microscopy (Fig. 3C).

### Incorporation of multiple recombinant capsids into HEV-LP by the co-infection of recombinant baculoviruses

**Figure 4:**
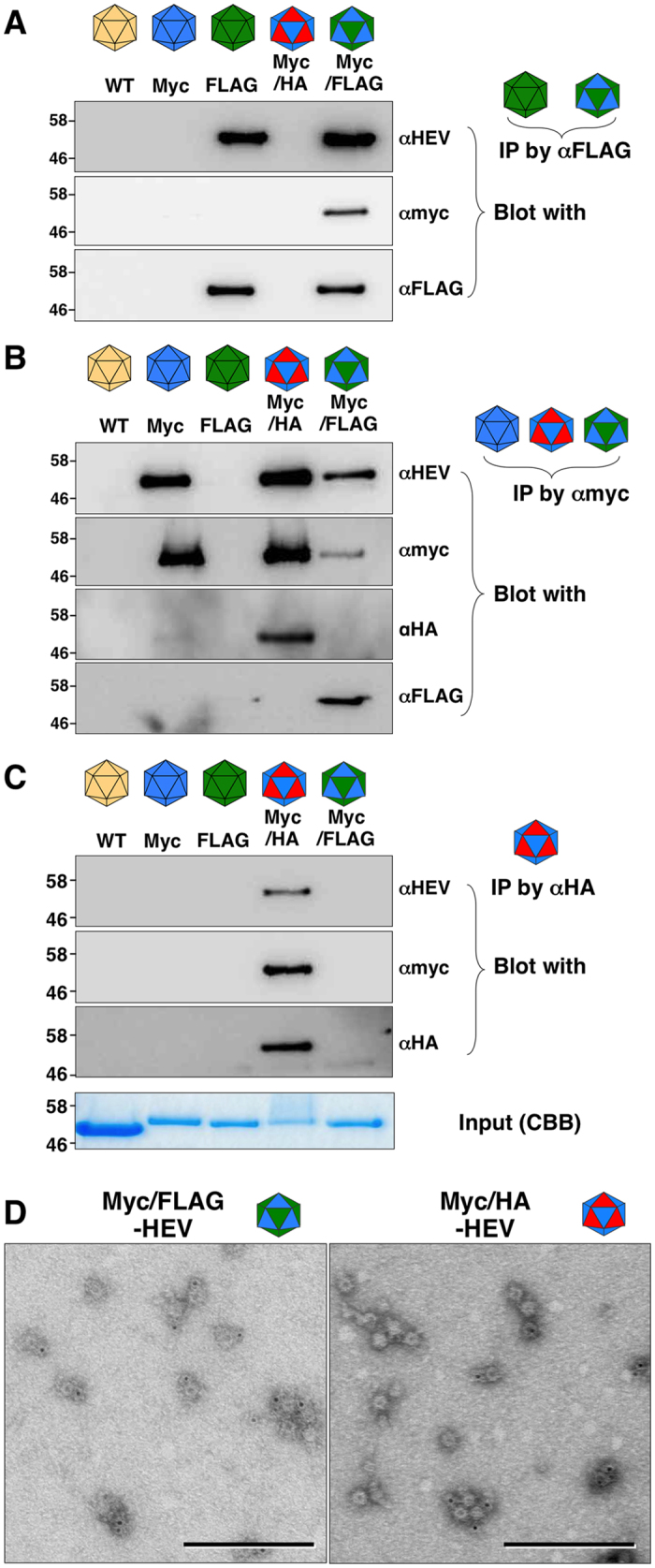
Incorporation of multiple recombinant capsids into HEV-LP. HEV-LP recovered from insect cells co-infected with recombinant viruses were immunoprecipitated by either anti-FLAG (**A**), -myc (**B**), or -HA (**C**) antibody and the immunoprecipitates were further examined by immunoblotting by indicated antibodies. In put of each HEV-LP stained by CBB is indicated in the bottom. (**D**) Immunoelectron microscopic observation of the chimeric HEV-LP consisting of the myc- and FLAG-tagged capsids and of the myc- and HA-tagged capsids were stained with anti-myc (left) and anti-HA antibody (right), respectively. Bars indicate 100 nm.

To confirm the incorporation of multiple recombinant capsids into HEV-LP, the HEV-LP were immunoprecipitated by either anti-FLAG, -myc, or -HA antibody and the immunoprecipitates were further examined by immunoblotting using the appropriate antibodies (Fig. 4). Immunoprecipitated with anti-FLAG antibody showed that HEV-LP consisting of the FLAG-tagged capsid alone and HEV-LP consisting of the FLAG- and myc-tagged capsids were co-precipitated, suggesting that the FLAG- and myc-tagged capsids were capable of being packaged into the same particles and both FLAG and myc epitopes were displayed on the surface (Fig. 4A). Moreover, not only HEV-LP consisting of the myc-tagged capsid alone but also those consisting of the myc- and HA-tagged capsids, or myc- and FLAG-tagged capsids, were co-precipitated by anti-myc antibody (Fig. 4B), and those consisting of the myc- and HA-tagged capsids were also co-precipitated by anti-HA antibody (Fig. 4C). In addition, the chimeric HEV-LP consisting of tagged capsids were examined by immunoelectron microscopy. By staining with anti-myc antibody, the HEV-LP consisting of the myc- and FLAG-tagged capsids were incorporated into the myc-tagged capsid. In addition, the HEV-LP consisting of the myc- and HA-tagged capsids were incorporated into the HA-tagged capsid (Fig. 4D). These results suggest that the HA-tagged HEV capsid protein alone is not able to form particles as shown in Fig. 2C, but is capable of being incorporated into the chimeric HEV-LP through interaction with the myc-tagged capsid protein.

### Production of chimeric HEV-LP bearing JEV-neutralizing epitopes

**Figure 5:**
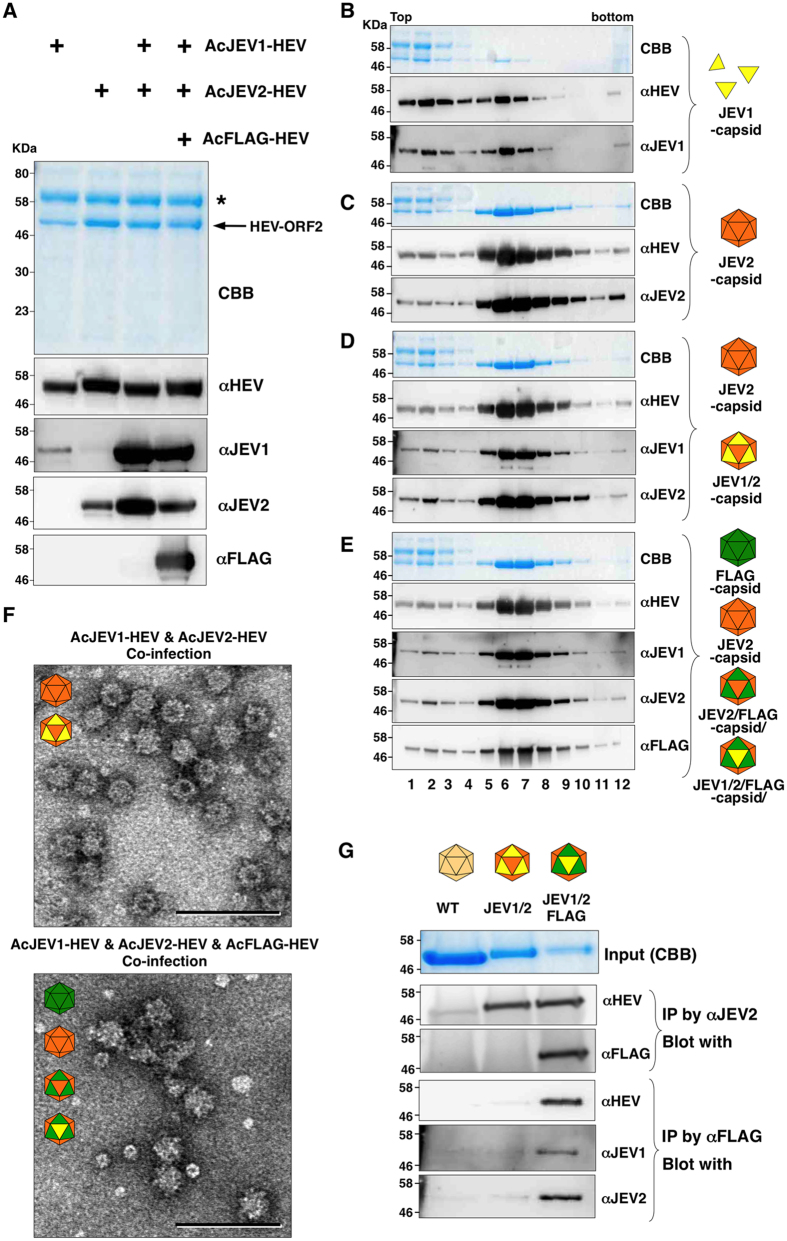
Production of chimeric HEV-LP bearing JEV neutralizing epitopes. (**A**) CBB staining and immunoblotting of the culture supernatants of Tn5 cells infected with various combination of AcJEV1-HEV (yellow), AcJEV2-HEV (orange) and AcFLAG-HEV (green). Asterisk and arrow in the CBB staining gel indicate bovine serum albumin and recombinant HEV capsids, respectively. (**B**~**E**) Sucrose density gradient fractionation of the culture supernatants of cells infected with either AcJEV1-HEV (**B**) or AcJEV2-HEV (**C**), co-infected with AcJEV1-HEV and AcJEV2-HEV (**D**), or co-infected with AcJEV1-HEV, AcJEV2-HEV, and AcFLAG-HEV (**E**). Putative recombinant and chimeric HEV-LP were indicated in the left of the panels. Each fractions were subjected to CBB staining and immunoblotting after SDS-PAGE. (**F**) Electron microscopic observation of the middle fractions of the sucrose density gradient. HEV-LP were imaged by negative staining. Bars indicate 100 nm. (**G**) Incorporation of multiple recombinant capsids into HEV-LP. HEV-LP recovered from insect cells infected with AcHEV (lane 1), co-infected with AcJEV1-HEV and AcJEV2-HEV (lane 2), and co-infected with AcJEV1-HEV, AcJEV2-HEV, and AcFLAG-HEV (lane 3) were immunoprecipitated by either anti-JEV2 or -FLAG antibody and the immunoprecipitates were further examined by immunoblotting by indicated antibodies. In put of each HEV-LP stained by CBB is indicated on the top.

We next attempted to present pathogen-derived epitopes on the surface of HEV-LP. We selected the neutralizing epitopes of JEV, JEV1 (337–345aa) and JEV2 (362–369aa) in the E protein^5^,^29^ (Fig. 1E) as the insertion peptides. HEV and JEV are human pathogens that utilize pigs as a reservoir and an amplifier, respectively. Therefore, the production of chimeric HEV-LP possessing JEV-neutralizing epitopes might be an ideal divalent vaccine for sterilizing pigs and as a consequence protect humans from potential infection by either virus. The recombinant capsid proteins were secreted in the culture supernatants and shown to retain the corresponding neutralizing epitopes by immunoblotting analyses (Fig. 5A). The sucrose density gradient ultra-centrifugation analyses revealed that a large portion of the HEV capsid protein bearing the JEV1 epitope was detected in the top fractions, and particle formation was inefficient (Fig. 5B), as seen in the HA-capsid protein, while that bearing the JEV2 epitope formed particles efficiently by a single expression (Fig. 5C). However, co-expression of these recombinant JEV-capsid proteins facilitated an efficient packaging of both proteins into chimeric HEV-LP (Fig. 5D), and the chimeric HEV-LP were detected by electron microscopy (Fig. 5F, upper panel).

In addition, triple expression of JEV1-, JEV2-, and FLAG-capsid proteins successfully yielded chimeric HEV-LP bearing JEV2- and FLAG-capsids or JEV1-, JEV2-, and FLAG-capsid proteins (Fig. 5A,E), and HEV-LP were detected in the fraction of the sucrose density gradients by electron microscopy (Fig. 5F, lower panel). To confirm the incorporation of the JEV1 epitope-capsid into the chimeric HEV-LP, the HEV-LP were immunoprecipitated with either anti-JEV2 or anti-FLAG antibody and the immunoprecipitates were further examined by immunoblotting using the appropriate antibodies (Fig. 5G). The JEV1-capsid protein was detected in both chimeric HEV-LP consisting of JEV1- and JEV2-capsids with or without FLAG-capsid, suggesting that the JEV1-epitope was packaged into the chimeric particles.

Collectively, these results suggest that co-expression of the recombinant HEV capsid proteins bearing foreign epitopes successfully yields the chimeric HEV-LP presenting multiple epitopes on the surface of the particles.

## Discussion

HEV-LP has potential not only as a mucosal vaccine antigen for hepatitis E but also as a mucosal vaccine carrier for various pathogens. In this study, we determined the insertion sites for foreign epitopes in the P domain of the HEV capsid protein based on the information of the crystal structure of HEV-LP described by us and others^24^,^30^. The co-expression of recombinant capsid proteins carrying the foreign epitopes resulted in the production of chimeric HEV-LP displaying multivalent foreign epitopes on the surface. The crystal structure of HEV-LP showed that the HEV capsid protein is composed of three functional domains, the S, M, and P domains^24^. Because HEV-LP crystal structure revealed that the S domain seems to form tightly packed icosahedral shells and the M domain is closely associated with the S domain, while the dimer of the P domain is shown to form a protrusion that participates in the cell-binding and have several disordered regions, suggesting that the P domain is suitable for the insertion of foreign epitopes^24^. Based on the HEV-LP crystal structure, we examined three insertion sites (amino acid residues 484 and 485, 488 and 489 and 555 and 556) located in the apical region of the large flexible loop within the P domain for the foreign epitopes. The expression of the recombinant capsid proteins harboring either a myc-, FLAG- or HA-tag varied depending on the insertion sites (Fig. 1B), and we selected the amino acid residues 488 and 489 of the large flexible loop on the protrusion for the insertion site. This loop is assumed to be not responsible for the particle formation via interaction with another copy of the capsid protein. Indeed, insertions of three out of five foreign peptides tested in this study (myc-tagged, FLAG-tagged and JEV2 epitope; Figs 2B and 5B) did not affect the particle formation. It was not clear why insertions of the remaining two peptides (HA-tagged and JEV1 epitope) impaired the particle formation in spite of the sufficient expression and secretion of the recombinant capsid protein. Jariyapong *et al*. previously reported the construction of chimeric HEV-LP by inserting the p18 peptide derived from gp120 of HIV-1 into the amino acid residues between 485 and 486, however expression levels of the chimeric HEV-LP were quite low^19^. Although we did not examine the immunogenicity of the chimeric HEV-LP in this study, the chimeric HEV-LPs by inserting the 11 peptide from herpes simplex virus into the in the C-terminus were capable of eliciting a specific antibody in mice upon oral administration^26^.

To our surprise, the particle formation of the capsid proteins harboring the HA-tagged and JEV1 epitope was restored by co-expression of the capsid proteins carrying the myc-tagged and JEV2 epitope, respectively. Although the mechanism underlying the generation of the chimeric HEV-LP was not clear, the expression of capsid proteins competent for particle formation together with those incompetent for particle formation clearly facilitated the production of chimeric VLP through the assembly of both capsid proteins. Indeed the construction of a chimeric VLP derived from various viral capsid proteins has been reported previously^31^, but this is the first report to show the possibility of rescuing the particle formation of the recombinant capsid proteins incompetent for particle formation by the co-expression of the competent ones. This co-expression method of recombinant HEV capsid proteins by a baculoviral system in insect cells expands the utility of HEV-LP as a platform to accommodate multiple foreign epitopes on the surface. HEV-LP is composed of 60 copies of the capsid protein, and if the recombinant capsids were randomly incorporated into the particles upon infection with the recombinant baculoviruses, the content of each capsid protein might be manipulated by the multiplicity of infection of each recombinant virus, and might be able to generate a chimeric HEV-LP carrying each epitope in the required ratio for a multivalent vaccine. Moreover, rescue of the particle formation of the assembly-deficient capsids by co-expression of the assembly-competent capsids might be applicable to the production of other VLP, including HIV-1, papillomavirus and influenza virus^32^,^33^,^34^. These chimeric VLP might be applicable not only for multivalent vaccines, but also as nanoparticle carriers for therapeutic use^19^. Zhao *et al*. reported the construction of chimeric VLPs of enterovirus 71 (EV71) possessing the neutralizing epitope of coxsackievirus A16 (CVA16). The chimeric VLPs were produced in yeast cells and protected mice from lethal challenge of CVA16^35^. Although this paper suggested that the chimeric VLPs have potential to be a vaccine against hand-foot-and-mouth disease (HFMD), it is obscure that insertion of other foreign epitopes can form VLPs. In contrast, we demonstrated that assembly-deficient capsids are incorporated into chimeric VLPs through co-expression with assembly-competent capsids.

In general, the production of chimeric VLP has relied mainly on two methods. The first is the insertion of target peptides or noncovalent bridging of foreign proteins by using biotin-streptavidin into either the N- or C- terminus^26^,^36^, and the other is chemical cross-linking^37^. The noncovalent method has a limitation in that foreign peptides cannot be readily accommodated into the VLP by using the bridge proteins, and it also has disadvantages for industrial use because of the immunogenicity of carrier proteins, the complicated process and the cross-linking method. However, based on the crystal structure information, the chimeric HEV-LP has several possible insertion sites into the surface region of VLP.

In a previous report, a chimeric HEV-LP possessing an integrin-binding RGD motif was suggested to enhance mucosal immune responses through an efficient uptake by the M-cells and to provide a non-invasive vector for cancer therapy through interaction with the overexpressing integrin receptors on the angiogenic endothelial cells^30^. In particular, the chimeric HEV-LP possessing a neutralization epitope of JEV is a promising candidate for a divalent mucosal vaccine for domestic pigs to eradicate infection of both HEV and JEV and thereby protect humans from potential infection with either virus. Although further studies including analyses of the immunogenicity and stability are needed, the chimeric HEV-LP bearing multiple epitopes generated in this study should provide important information toward the development of novel mucosal vaccines.

## Additional Information

**How to cite this article**: Shima, R. *et al*. Production of hepatitis E virus-like particles presenting multiple foreign epitopes by co-infection of recombinant baculoviruses. *Sci. Rep.* **6**, 21638; doi: 10.1038/srep21638 (2016).

## Figures and Tables

**Table 1:** Primers used in this study.

Primer	Primer sequence
HEV(334)/BamHI	5′-CCCGGATCCATGGCTACATCACCAGCCCCTGA-3′
HEV(1452)-c-myc/Rv	5′-CAGGTCCTCCTCTGAGATCAGCTTCTGCTCCGTGGTCTGGTCGTACTCAG-3′
HEV(1452)-HA/Rv	5′-AGCGTAATCTGGAACATCGTATGGGTACGTGGTCTGGTCGTACTCAG-3′
HEV(1452)-FLAG/Rv	5′-CTTGTCGTCATCGTCTTTGTAGTCCGTGGTCTGGTCGTACTCAG-3′
HEV(1464)-c-myc/Rv	5′-CAGGTCCTCCTCTGAGATCAGCTTCTGCTCGGACGACCCATACGTGGTCT-3′
HEV(1464)-HA/Rv	5′-AGCGTAATCTGGAACATCGTATGGGTAGGACGACCCATACGTGGTCT-3′
HEV(1464)-FLAG/Rv	5′-CTTGTCGTCATCGTCTTTGTAGTCGGACGACCCATACGTGGTCT-3′
HEV(1464)-JE(337)/Rv	5′-GAGGCTCGCAACGGAGACAATCGGAATGGACGACCCATACGTGGTCT-3′
HEV(1464)-JE(362)/Rv	5′-CTTTGAATTGGCACTGGAAGTCGCGGACGACCCATACGTGGTCT-3′
HEV(1665)-c-myc/Rv	5′-CAGGTCCTCCTCTGAGATCAGCTTCTGCTCGGCCTTAGTCGTGCTAGCCT-3′
HEV(1665)-HA/Rv	5′-AGCGTAATCTGGAACATCGTATGGGTAGGCCTTAGTCGTGCTAGCCT-3′
HEV(1665)-FLAG/Rv	5′-CTTGTCGTCATCGTCTTTGTAGTCGGCCTTAGTCGTGCTAGCCT-3′
c-myc-HEV(1453)/Fw	5′-GAGCAGAAGCTGATCTCAGAGGAGGACCTGTATGGGTCGTCCACCAACCC-3′
HA-HEV(1453)/Fw	5′-TACCCATACGATGTTCCAGATTACGCTTATGGGTCGTCCACCAACCC-3′
FLAG-HEV(1453)/Fw	5′-GACTACAAAGACGATGACGACAAGTATGGGTCGTCCACCAACCC-3′-3′
c-myc-HEV(1465)/Fw	5′-GAGCAGAAGCTGATCTCAGAGGAGGACCTGACCAACCCCATGTATGTCTC-3′
HA-HEV(1465)/Fw	5′-TACCCATACGATGTTCCAGATTACGCTACCAACCCCATGTATGTCTC-3′
FLAG-HEV(1465)/Fw	5′-GACTACAAAGACGATGACGACAAGACCAACCCCATGTATGTCTC-3′
JE(337)-HEV(1465)/Fw	5′-ATTCCGATTGTCTCCGTTGCGAGCCTCACCAACCCCATGTATGTCTC-3′
JE(362)-HEV(1465)/Fw	5′-GCGACTTCCAGTGCCAATTCAAAGACCAACCCCATGTATGTCTC-3′
c-myc-HEV(1666)/Fw	5′-GAGCAGAAGCTGATCTCAGAGGAGGACCTGGGCTACCCGTATAACTATAA-3′
HA-HEV(1666)/Fw	5′-TACCCATACGATGTTCCAGATTACGCTGGCTACCCGTATAACTATAA-3′
FLAG-HEV(1666)/Fw	5′-GACTACAAAGACGATGACGACAAGGGCTACCCGTATAACTATAA-3′
HEV(1824)XhoI/Rv	5′-GGGCTCGAGTTAGGCAAGGGCCGAATGTGGGGC-3′
